# Patient Experience of a Student‐Led Rural Indigenous Outplacement Dental Clinic

**DOI:** 10.1111/ajr.70007

**Published:** 2025-03-03

**Authors:** Sandra March, Clare Mangoyana, Ratilal Lalloo, Laurence J. Walsh

**Affiliations:** ^1^ The University of Queensland Brisbane Queensland Australia

**Keywords:** Indigenous health service—university partnership, Indigenous oral health, oral healthcare access

## Abstract

**Objective:**

The study aimed to explore the patient view of care provided by a student‐led rural Indigenous dental clinic and of service access.

**Methods:**

Clinic patients voluntarily undertook a short exit survey on completion of their care. Participants were asked how they initially knew about the service and the location from which they had travelled to attend. A 5‐point Likert scale ranging from Very Satisfied to Very Dissatisfied measured patient satisfaction with the dental clinic services.

**Results:**

Participants (*n* = 159) age ranged from 18 years, with 49% (78/159) aged over 55, while 60% (96/159) were female. Indigenous status was identified by 48% (77/159) of survey participants. Referral by the local Indigenous Health Service was the primary means of introduction to the dental clinic for patients, with word of mouth second. Patients travelling from outside the local area accounted for 41% (66/159) of dental clinic clientele. An overwhelming 97.5% (155/159) ‘Very Satisfied’ response by participants indicated their unqualified acceptance and approval of the quality of student‐led provision of oral healthcare services.

**Conclusion:**

Patients highly rated the students' oral healthcare provision. The clinic's presence greatly facilitated access to dental care for local area residents and Indigenous clients living geographically much farther afield.

**Summary**

- What is already known on this subject
  ○ By all indicators, Indigenous Australians have much poorer oral health than their non‐Indigenous counterparts.
  ○ Personal and socioeconomic factors restrict the provision of oral healthcare and its utilisation by Indigenous Australians, geographical access to oral healthcare being a particular challenge for rural communities.
  ○ Culturally appropriate settings enhance Indigenous utilisation of health care provision.
- What this study adds to our knowledge
  ○ A partnership arrangement between a university and an Indigenous Health Service greatly enhances accessibility to oral healthcare for a rural Indigenous community.
  ○ Patients are prepared to travel considerable distances to attend the health service–embedded student‐led outplacement dental clinic.
  ○ Local patients and those travelling from afar much appreciate the service and are overwhelmingly satisfied with the quality of oral healthcare provided to them by dental students.

## 1 Introduction

Disparities in oral healthcare provision and utilisation in Australia result in adverse outcomes for Indigenous and rural and remote communities [1, 2]. Inadequate rural oral health workforce capacity and geographical factors have served to magnify the problem [3]. Successful initiatives addressing these issues include development by universities of dental student outplacement opportunities in rural Indigenous communities [4]. Enhanced authentic student learning experiences provided by such outplacements enable better access to oral healthcare for the community served [5].

The University of Queensland (UQ) School of Dentistry partnered in 2013 with Goondir Health Services (GHS), an Aboriginal and Torres Strait Islander Community Controlled Health Service (ATSICCHS), to jointly establish a student‐led outplacement dental clinic situated in the South‐West Queensland rural community of Dalby. Supervised groups of four final‐year UQ dental students complete 8‐ or 11‐week clinical rotations providing free preventive and therapeutic oral health services to the community.

This study, part of a project researching oral health, social, economic and educational outcomes of the embedded student‐led outplacement dental clinic in Dalby [5, 6], aimed to explore clinic accessibility and patients' views on service quality.

## 2 Methods

### 2.1 Ethics Approval

Institutional Human Research Ethics approval for this study was granted by the University of Queensland Human Research Ethics Committee, Approval number 2019001480.

### 2.2 Context

During an 11‐week rural Indigenous clinic outplacement rotation from mid‐August until early November 2019, four UQ final year dental students (three female, one male; two domestic, two international) provided oral health services during 370 separate patient interactions. Categorised by item code [7], 724 individual oral health services completed by these students included 68% (491/724) diagnostic and preventive, 27% (194/724) restorative tooth preservation and 5% (37/724) tooth extraction and replacement. Clinic records indicated that GHS clients attended 71% (261/370) of the 370 appointments.

### 2.3 Study Design and Participants

On completion of their current course of dental care, Dalby outplacement clinic patients seen between August and November 2019 were invited by the senior clinic support staff member to participate in a short exit survey (Appendix S1). Participants were asked how they knew about the clinic, how far they had travelled to access it and how satisfied they were with the services provided by students. Satisfaction was measured utilising a 5‐point Likert‐type scale ranging from Very Satisfied to Very Dissatisfied.

### 2.4 Ethical Considerations

Prior to project commencement, the metropolitan‐based research team visited Dalby to consult personally with GHS management and staff to seek their collaboration and contributions. GHS management expressed written support for the study aims, welcoming the research initiatives. GHS staff advised on the most culturally appropriate and effective approaches to data collection. They suggested that a locally resident Senior Dental Assistant personally well known to many clients, administer the concise paper‐based tick‐box questionnaire. Patients were assured verbally and in print that their participation was voluntary, entirely confidential, that they could withdraw from the study at any time and that the decision whether to participate or not would not affect any existing or future relationship with GHS or UQ.

## 3 Results

**FIGURE 1:**
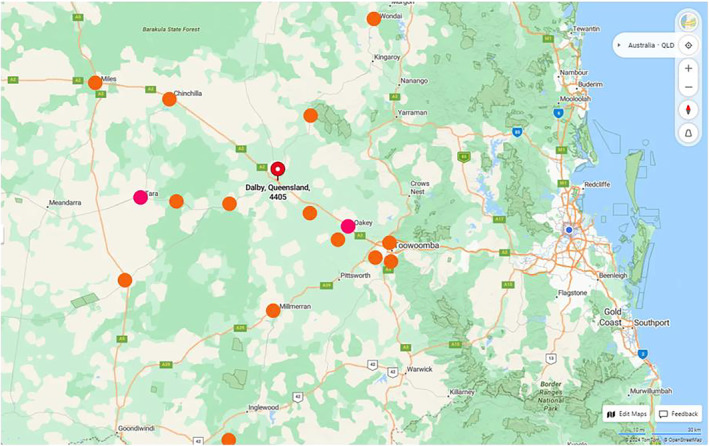
Distances travelled by patients attending dental clinic. †Pink dots denote locations where patient transport is provided. Orange dots denote other locations from which patients travelled. ‡Distance scale: ‐‐‐‐‐ 10 miles ‐‐‐‐‐‐‐‐‐ 30 km.

Of 161 completed surveys, two responses from participants aged < 18 years were excluded. The remaining demographic (*n* = 159) was 60% female and 40% male. Indigenous status was identified by 48% of respondents and 49% were older than 55 years. Participants indicated that the most common prompt for clinic attendance was GHS referral (56% or 89/159), followed by word‐of‐mouth endorsement (40% or 64/159). Only three (2%) respondents cited advertising as their means of knowledge of the clinic. While 59% (93/159) of patients reported being from Dalby and local surrounds, 41% (66/159) indicated that they had travelled from 17 other towns located outside the district to access the dental clinic (Figure 1 and Table 1). Participant responses related to satisfaction with the oral health services provided by the student‐led dental clinic revealed 97.5% (155/159) were Very Satisfied, 2% (3/159) were Satisfied and only 0.5% (1/159) was Very Dissatisfied.

**TABLE 1:** Distance and travel time for dental clinic patients.

Town	Distance: kilometres	Travel time: minutes	Patient numbers
Yelarbon	199.4	133	1
Wyreema	90.5	64	4
Wondai	141.0	102	1
Weranga	67.9	49	1
Toowoomba	83.2	64	2
Tara	89.5	59	8
Oakey	56.9	39	24
Moonie	133.0	92	1
Millmerran	89.7	63	1
Miles	128.0	94	1
Kumbarilla	39.3	30	2
Kingsthorpe	70.0	49	1
Jondaryan	40.6	28	1
Chinchilla	82.3	59	1
Canaga	80.2	54	1
Bell	40.2	29	3
Aubigny	61.7	46	1
Other—not specified			12
Dalby local area	< 20.0	< 20	93
			159

*Note:* Location, distance and travel time to attend Dalby student‐led outplacement dental clinic.

## 4 Discussion

Aboriginal and Torres Strait Islander Community Controlled Health Services have positively impacted the health of Indigenous people [8]. These independent non‐profit organisations provide culturally competent, holistic primary health care and medical services as well as promoting health through education of their clients. GHS serves around 4000 Indigenous clients from Dalby in South‐East Queensland to St George in South‐West Queensland, an area of 72 000 km^2^. Embedded dental clinics within the Dalby (2013) and St George (2017) premises have improved Indigenous health outcomes by increasing accessibility to and utilisation of culturally appropriate oral health care services [9]. As holistic healthcare policy, GHS clients are routinely referred by medical staff to the student‐led dental clinic for oral health assessment [10], explaining the most common means of finding out about the clinic being GHS referral (56%), followed closely by word‐of‐mouth (40%). While 48% of survey participants identified as Indigenous, Goondir clients comprised 71% of patient interactions during the survey period.

More remotely located (515 km inland) in a community of around 3000 with only part‐time private and government dental service availability, the student‐led dental clinic in St George has considerably enhanced accessibility and utilisation of oral healthcare. Over 2 years between mid‐2017 and mid‐2019, 60 dental students undertook 2973 free‐of‐charge patient interactions, 77% with GHS clients. Patients identifying as Indigenous accounted for 65% of these visits. With a larger population (12 700) and situated only 215 km inland, Dalby boasts several private dental practices and a government dental clinic. The additional access to oral healthcare provided free of charge by that student‐led dental clinic has been found to benefit community health, had a positive economic [6] impact while fulfilling university educational goals [11, 12] and meeting professional accreditation requirements [13].

At the time of the study, GHS provided regular free client transport to and from two nearby communities: Tara and Oakey. Unsurprisingly, residents of these two communities made up 16.4% of survey respondents as they were able to conveniently access the multiple health services offered in one place at one time. Demonstrating the high degree of trust and regard held by GHS clients for the dental clinic, one client reported a round trip of 400 km to attend appointments. Trust developed by the clinic's situation within the culturally safe GHS environs of ‘our mob’ greatly enhanced Indigenous community utilisation of the oral health services. Stated high levels of satisfaction with the clinic's services confirmed that trust. Prior aspects of our research revealed that patients were pleased that students spent plenty of time communicating and explaining treatment and did not rush, suiting Indigenous cultural norms [5]. Clinic patients also felt mutual benefit arose from their positive role in assisting student learning in areas of appropriate communication and clinical practice [5]. The high degree of satisfaction expressed by patients may also have stemmed from their feelings about the clinic's heavy focus (95%) on health positive preventive and restorative services compared with less desirable tooth loss and replacement treatments.

The exit survey was formatted and delivered following Indigenous GHS staff advice. However, the survey's brevity meant specific satisfaction themes were unexplored. A bias toward the extremely high number of very positive satisfaction responses because of the timing and personal delivery of the survey by clinic staff cannot be discounted.

## 5 Conclusions

Utilisation of culturally appropriate oral healthcare by an Indigenous community was greatly enhanced by easy access to a student‐led outplacement dental clinic situated within a holistic Indigenous health service premises. The mutually beneficial university–Indigenous health service partnership enabled free oral healthcare delivery to disadvantaged rural communities. Dental clinic patients overwhelmingly expressed satisfaction with the care provided by the student operators.

## Author Contributions

**Sandra March:** conceptualization, funding acquisition, writing – original draft, methodology, supervision, project administration, visualization. **Clare Mangoyana:** data curation, formal analysis, visualization, writing – review and editing, investigation, resources. **Ratilal Lalloo:** conceptualization, funding acquisition, writing – review and editing. **Laurence J. Walsh:** conceptualization, funding acquisition, writing – review and editing.

## Ethics Statement

Institutional Human Research Ethics approval for this study was granted by the University of Queensland Human Research Ethics Committee B, Approval number 2019001480.

## Conflicts of Interest

The authors declare no conflicts of interest.

## Supporting information


Appendix S1

## Data Availability

The data that support the findings of this study are available on request from the corresponding author. The data are not publicly available due to privacy or ethical restrictions.
