# Supplementary material for: Patient Experience of a Student‐Led Rural Indigenous Outplacement Dental Clinic
Source: Aust J Rural Health. 2025 Mar 3;33(2):e70007. doi: 10.1111/ajr.70007 (PMC11874262; doi:10.1111/ajr.70007)
Supplement: Supplementary file 1 — Appendix S1 [file AJR-33-0-s001.docx]

**APPENDIX:**

***Indigenous community benefits of a student Outplacement Dental Clinic***

**Study Title: *Outcomes of clinical placement: an audit of the health and social benefits of a student dental clinical outplacement program for an Indigenous population in a rural Queensland community.***

As you have now completed your current course of care here at the UQ Dalby Dental Clinic, you are invited to participate in a short (less than five minutes) survey to explore your views on the quality of care you have received from the dental students and access to this service.

- **Please tick the box to indicate you have read and understood the Participant Information provided and consent to participate in this survey**

| Are you Male/Female? | - **Male** - **Female** - **Prefer not to say** |
| --- | --- |
| Do you identify as Aboriginal or Torres Strait Islander | - **Yes** - **No** |
| Age group | - **18-24** - **25-34** - **35-44** - **45-54** - **55+** |
| How did you find out about the dental clinic? | - **Advertisement** - **Word of mouth** - **Referral** - **Other** (please specify below) …………………………………………. |
| How far did you travel to access the clinic? | - **Local Dalby area** - **Other** (Please specify below)   ………………………………………… |
| How satisfied were you with this service? | - **Very satisfied** - **Satisfied** - **Neither satisfied nor dissatisfied** - **Dissatisfied** - **Very dissatisfied** |
